# Papillary thyroid carcinoma: 6 cases from 2 families with associated lymphocytic thyroiditis harbouring RET/PTC rearrangements

**DOI:** 10.1054/bjoc.2001.2187

**Published:** 2001-12

**Authors:** C Mechler, A Bounacer, H Suarez, M Saint Frison, C Magois, G Aillet, A Gaulier

**Affiliations:** Department of Pathology, C.H. Victor Dupouy, Argenteuil cedex, 95107, France; Laboratoire d'instabilité génétique et de cancer (UPR 2169 CNRS), Villejuif cedex, 94801, France; Department of pathology, C.H.R.U. of Nantes, Nantes Cedex, 44035, 01, France

**Keywords:** familial PTC, lymphocytic thyroiditis, ret oncogene activation

## Abstract

Familial papillary thyroid carcinoma (PTC) is a well recognized disease. However, genetic predisposition to familial PTC is rare and the molecular alterations at the origin of the pathology are unknown. The association between PTC and lymphocytic thyroiditis (LT) has been reported recently. We communicate here 6 cases of PTC associated with LT in 2 unrelated families. PTC was diagnosed on classical nuclear and architectural criteria. It was bilateral in 5 cases. Architecture was equally distributed between typical PTC and its follicular variant. LT was present in variable degrees, including in 4 cases, oncocytic metaplasia. Using the RT-PCR technique, we observed a RET/PTC rearrangement in the carcinomatous areas of patients of both families: PTC1 in family 1 and PTC3 in family 2 and a RET/PTC rearrangement in non-malignant thyroid tissue with LT in family 2. The RET/PTC band was weaker or absent in pure LT areas. Furthermore, using a polyclonal *ret* antibody, an apical or a diffuse cytoplasmic *ret* onc protein immunolabelling was observed in the three patients with RET/PTC1 rearrangement and in the three patients with RET/PTC3 rearrangement. In conclusion our data: (1) show the presence of a RET/PTC 1 or 3 rearrangement (depending on the family) together with a variable expression of *ret* protein in all the PTCs; (2) suggest that the molecular event at the origin of the PTCs seems to be particular to each one of the studied families; and (3) confirm that the *ret* proto-oncogene activating rearrangement(s) is an early event in the thyroid tumorigenic process and that it can be observed in association with LT. © 2001 Cancer Research Campaign http://www.bjcancer.com


*British Journal of Cancer* (2001) **85**(12), 1831–1837
© 2001 Cancer Research Campaign
doi: 10.1054/ bjoc.2001.2187, available online at http://www.idealibrary.com on IDEAL
http://www.bjcancer.com

### Papillary thyroid carcinoma: 6 cases from 2 families with associated lymphocytic thyroiditis harbouring RET/PTC rearrangements

**C Mechler[1,a], A Bounacer[2,a,b], H Suarez[2], M Saint Frison[1], C Magois[3], G Aillet[3] and A Gaulier[1]**

[1]Department of Pathology, C.H. Victor Dupouy, 95107 Argenteuil cedex, France; [2]Laboratoire d'instabilité génétique et de cancer (UPR 2169 CNRS), 94801 Villejuif cedex, France; [3]Department of pathology C.H.R.U. of Nantes, 44035 Nantes Cedex, 01, France

**Summary** Familial papillary thyroid carcinoma (PTC) is a well recognized disease. However, genetic predisposition to familial PTC is rare and the molecular alterations at the origin of the pathology are unknown. The association between PTC and lymphocytic thyroiditis (LT) has been reported recently. We communicate here 6 cases of PTC associated with LT in 2 unrelated families. PTC was diagnosed on classical nuclear and architectural criteria. It was bilateral in 5 cases. Architecture was equally distributed between typical PTC and its follicular variant. LT was present in variable degrees, including in 4 cases, oncocytic metaplasia. Using the RT-PCR technique, we observed a RET/PTC rearrangement in the carcinomatous areas of patients of both families: PTC1 in family 1 and PTC3 in family 2 and a RET/PTC rearrangement in non-malignant thyroid tissue with LT in family 2. The RET/PTC band was weaker or absent in pure LT areas. Furthermore, using a polyclonal *ret* antibody, an apical or a diffuse cytoplasmic *ret* onc protein immunolabelling was observed in the three patients with RET/PTC1 rearrangement and in the three patients with RET/PTC3 rearrangement. In conclusion our data: (1) show the presence of a RET/PTC 1 or 3 rearrangement (depending on the family) together with a variable expression of *ret* protein in all the PTCs; (2) suggest that the molecular event at the origin of the PTCs seems to be particular to each one of the studied families; and (3) confirm that the *ret* proto-oncogene activating rearrangement(s) is an early event in the thyroid tumorigenic process and that it can be observed in association with LT. © 2001 Cancer Research Campaign http://www.bjcancer.com

**Keywords**: familial PTC; lymphocytic thyroiditis; ret oncogene activation

Papillary thyroid carcinoma (PTC), sometimes found as a familial disease (Fisher et al, 1989; Lote et al, 1980; Ron et al, 1991; Gorson, 1992), may be occasionally associated with Graves' disease (Soh and Parc, 1993), lymphocytic thyroiditis (McKee et al, 1993; Okayasu et al, 1995) or Hashimoto's thyroiditis (Walker et Paloyan, 1990; Sclafani et al, 1993; Reda et al, 1997; Wirtschafter et al, 1997). It has been suggested that lymphocytic thyroiditis may be a predisposing pathology for PTC, perhaps through the activation of the *ret* proto-oncogene by a mechanism of chromosomal rearrangement (Ishizaka et al, 1991; Wirtschafter et al, 1997). However causative relations between the 2 nosological entities are still uncertain.

The *ret* proto-oncogene, located on chromosome 10q 11.2, encodes for a protein structurally related to transmembrane receptors with a tyrosine kinase (TK) domain (Takahasi et al, 1988), and recently it has been shown that its putative ligands are the Glial-cell-line Derived Neurotrophic Factor (GDNF), neurturin, artemin, enovin and persephin (Suárez, 1998a, b; Jhiang, 2000). The gene is expressed in a variety of neuronal cell lineages, as well as in the kidney and enteric nervous system (Suárez, 1998a). The activated *ret* oncogene was first isolated by the transfection of NIH-3T3 cells with DNA from a human T cell lymphoma (Takahashi et al, 1988). Germline point mutations of *ret* have been described in patients afflicted by MEN 2A and MEN 2B syndromes or familial medullary thyroid carcinoma (FMTC), indicating an involvement of *ret* in the pathogenesis of these diseases (Chappuis et al, 1996; Bounacer et al, 1997). In 'spontaneous' or radiation-associated PTCs, 8 forms of the *ret* proto-oncogene activated by rearrangement have been identified and designated RET/PTC1 to RET/PTC8 (Jhiang, 2000; Klugbauer et al, 2000). All the activated forms of the proto-oncogene are the consequence of specific oncogenic rearrangements fusioning the TK domain of *ret* with the 5′ domain of different genes (Suárez, 1998b; Klugbauer and Rabes, 1999). The RET/PTC activating rearrangements of the *ret* proto-oncogene are with *ras*-activating point mutation and *trk*-activating rearrangements, the most frequent genetic alteration observed in PTCs (Suárez, 1998a, b).

Indeed, contrasting with the high and very similar frequency (about 60%) of activation by rearrangement observed in the therapeutic or accidental radiation-associated tumours (Bounacer et al, 1997), the frequency of *ret* activation by this mechanism in 'spontaneous' thyroid tumours varies widely between different studies: from 2.5% to 34% (Bongarzone et al, 1989, 1993; Grieco et al, 1990; Santoro et al, 1994; Delvincourt et al, 1996; Bounacer et al, 1997). It has been postulated that this variation observed in 'spontaneous' tumours, could be the result of the different geographical origins of populations studied, the age at tumour occurrence, or the sensitivity of the experimental methods used to detect the rearrangement. All the RET/PTC oncogenic forms of *ret*, seem to

*Received 19 January 2001*
*Revised 2 August 2001*
*Accepted 18 September 2001*

*Correspondence to:* C Mechler

[a]Charlotte Mechler and Ali Bounacer should be considered co-first authors.
[b]Present address: Department of Pathology, University of Wales College of Medicine Heath Park, Cardiff, CF4 4XN UK.

be specific of PTCs. However, the chimeric transcript of RET/PTC1 oncogene, has also been found in one study, in radiation-associated follicular adenomas (Bounacer et al, 1997).

In the present report we studied one follicular adenoma and PTCs obtained from members of 2 unrelated families in which we suspected a familial predisposition to develop such conditions. The tumours were associated in these patients with thyroiditis and Hashimoto's thyroiditis. In order to look for the eventual presence of RET/PTC chimeric genes in the tumours obtained from the different members of the families and to try to verify if the familial predisposition for PTC could be attributed to a particular and unique RET/PTC rearrangement (germinal?), we have studied: (1) by RT-PCR, mRNAs prepared from paraffin-embedded tissues obtained from tumoural, thyroiditis and normal thyroid tissue areas and from peripheral lymphocytes of members of one of the families; and (2) by immunohistochemistry, the eventual presence and localization in these tissues of the *ret* onc protein.

### MATERIALS AND METHODS

### Patients

Both family trees are reported in Figure 1. Clinical, pathological and molecular findings are summarized in Table 1.

In the first family, case 1 (53 years), case 2 (27 years) and case 3 (19 years) underwent total thyroidectomy. The histological diagnoses were a bilateral follicular and trabecular non-encapsulated PTC in case 1, a bilateral diffuse sclerosing variant of PTC in case 2, and a PTC with one metastatic lymph node in case 3. The patient in case 4 (32 years) underwent a partial thyroidectomy for a nodule, which was a follicular adenoma.

In the second family, case 5 (67 years), case 6 (42 years) and case 7 (41 years) underwent total thyroidectomy for a bilateral encapsulated PTC with 3 metastatic lymph nodes in case 5, and a bilateral encapsulated PTC in case 6. In case 7 one lobe presented a non-encapsulated papillary microcarcinoma, and the other 3 microfollicular PTCs.

LT was intense in cases 1, 2, 3, 4 and 7. The lymphoid infiltrate penetrates the carcinomatous areas.

As controls we studied 4 sporadic PTCs.

After a 10-year follow up, no recurrence of the PTCs occurred in any patient of family 1 or 2.

All thyroid specimens were studied macroscopically and fixed in neutral formalin (cases 1, 2, 3, 4) or Dubosq Brazil (cases 5, 6, 7) after

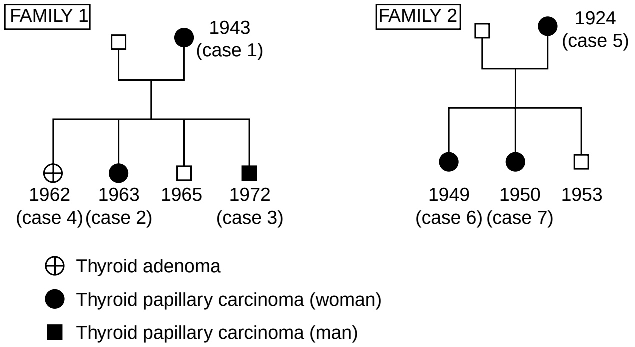

**Figure 1** Genealogical trees from families 1 and 2

**Table 1** Pathological, molecular and immunohistological study of the different samples

| Family | Cases | Age at tumor Diagnosis | Histology | Lymphocytic thyroiditis[a] | ret rearrangement | | ret expression | | | |
|---|---|---|---|---|---|---|---|---|---|---|
| | | | | | | | Transcription (RT-PCR)[e] | | Transduction (IHC)[c] | |
| | | | | | Tumour | Thyroiditis | Tumour | Thyroiditis | Tumour | Thyroiditis |
| 1 | No. 1 | 53 years | PTC | +++ | RET/PTC1 | – | + | +/– | C + | C +/– |
| | No. 2 | 27 years | PTC | +++ | RET/PTC1 | – | + | +/– | C + | C +/– |
| | No. 3 | 19 years | PTC | +++ | RET/PTC1 | – | + | +/– | C + | C +/– |
| | No. 4 | 28 years | adenoma | +++ | – | – | – | – | – | – |
| 2 | No. 5 | 67 years | PTC | + | RET/PTC3 | RET/PTC3 | + | + | A ++ | A +/– |
| | No. 6 | 42 years | PTC | + | RET/PTC3 | RET/PTC3 | + | + | A + | A +/– C +/– |
| | No. 7 | 41 years | PTC | +++ | RET/PTC3 | RET/PTC3 | + | + | A ++ | A +/– C +/– |

[a]Lymphoid infiltration in non neoplastic parenchyma is given as poorly distributed foci +, diffuse with low density ++, diffuse and abundant +++. [b]Reverse transcription-polymerase chain reaction. [c]Immunohistochemistry: localization of *ret* labelling: C = cytoplasmic diffuse; A = apical.

*© 2001 Cancer Research Campaign*

a 48-hour immersion. All the resected material was paraffin embedded and studied with haematoxylin and eosin safranin staining.

### RNA extraction from fixed thyroid tissue samples

RNA isolation from paraffin-embedded tissue samples was performed according to a previously described procedure (Bounacer et al, 1997). Briefly, 2 20 µm tissue sections were cut from blocks of paraffin-embedded tissue and deparaffinized in 2 serial washes of 200 µl of xylene in 0.5 ml tubes. After centrifugation (12 000 ***g*** for 15 min), the pellet was washed twice with 200 µl of ethanol and dried. The pellet was resuspended in 100 µl of 50 mM Tris pH 8.6, 1 mM EDTA, 0.5% Tween, 80 U rRNasin Ribonuclease inhibitor (Promega), 10 U RQ1 RNase free DNAse (Promega) and 1 M KCl containing 0.3 mg $ml^{-1}$ proteinase K (Merck), and incubated for 3 h at 37˚C and 10 min at 95˚C. After centrifugation (12 000 ***g*** for 15 min at 4˚C), the supernatant was transferred to a 0.5 ml tube and precipitated at –20˚C overnight with 1:10 volume of 3 M sodium acetate and 3 volumes of ethanol. After centrifugation, the pellet was washed with 70% ethanol, air dried and resuspended in 40 µl of sterilized water.

RNAs from peripheral blood lymphocytes were prepared using a Su total RNA isolation kit, according to the protocol of the manufacturer (Promega).

### RT-PCR method for detecting RET/PTC oncogenes, ret gene expression and β-actin gene as a reporter target for amplification

The reverse transcription (RT) reaction was performed with 8 µl of RNA extracted from paraffin-embedded tissue extracts. The RNA was reverse transcribed by incubation for 1 h at 37˚C in a final volume of 40 µl containing 1000 U MMLV reverse transcriptase (Gibco, Brl), 1 × RT buffer (50 mM Tris-HCl pH 8.3, 75 mM Kcl, 3 mM Mg $Cl_2$), 1 mM of each dNTP (Pharmacia), 4 mM DTT (Gibco, Brl), 1 U rRNAsin Ribonuclease Inhibitor (Promega) and 500 ng of Random hexamers (Pharmacia). The reaction was stopped by incubation for 5 min at 95˚C. One quarter of the cDNA (10 µl), was used for PCR amplification with outer primers. A second round of PCR was done with nested primers using 1:10 of the first round PCR product.

The PCR amplifications were performed using an automatic thermocycler (Perkin-Elmer), in the presence of 2 U of Taq polymerase (Ampli Taq, Perkin-Elmer, Cetus), 30 pmol of each sense and antisense primers, 1 × reaction buffer (10 mM Tris-HCl pH 8.3, 5 mM KCl, 0.01% gelatine), 250 µM of each dNTP and 1.5 mM $MgCl_2$ in a total volume of 50 µl. For RET/PTC oncogenes and β-actin, PCR reaction consisted of one cycle at 94˚C for 2 min followed by 40 cycles of denaturation, annealing and extension at 94˚C (30 s), 60˚C (1 min) and 72˚C (1 min) respectively. After 10 min at 72˚C to ensure that the final extension step was complete, aliquots (10 µl) of the products of the reaction were analysed by electrophoresis through a 2% agarose gel. PCR primer sequences used to study the RET/PTC are given in Table 2. The primers to study β-actin were: forward: 5′-ctcttgctctgggcctcgtc-3′ and reverse: 5′-ctcttgctctgggcctcgtc-3′. To study the expression in the different tissues of the TK domain of the RET gene, the PCR reaction consisted of 35 cycles of amplification at 95˚C for 30 s, 55˚C for 30 s, and 72˚C for 1 min and the primers were out-nested primers: 5′-caccggatggagaggccagacaactgcagc-3′ forward and 5′-accggccttttgtccggctc-3′ reverse. 5 µl of the reaction product were amplified in a second round PCR reaction using the in-nested primers 5′-gagaggccagacaactgcagc-3′ forward and 5′-ccttttgtccggctcctgcttccagcattg-3′ reverse. These primers correspond to exon 16, 5′ upstream and exon 17, 3′ downstream spanning a 1150 bp intron of *ret*. Precautions were taken to prevent PCR contamination. Indeed, in each experiment RNA-negative samples, or samples containing normal RNA prepared from normal thyroid tissue without C cells, were run in parallel.

### Immunohistochemical study

The immunohistochemical study was performed with a polyclonal antithyrocalcitonin antibody (Ab) (Dako 1/50), and a *ret* polyclonal anti-TK domain Ab (C-19)G from Santa Cruz.

The different tests were carried out with an LSAB (R)2 peroxidase kit (Dako) according to a previously described procedure (Gaulier et al, 1994) or a Ventana automated procedure. The slide pretreatment included heating with a pressure cooker in EDTA pH 7 and incubation in 40% formic acid (20 min). The final working dilution for the polyclonal *ret* Ab was 1/200.

Positive control was carried out by labelling ganglion cells on human appendix sections with *ret* Ab. Negative control for the immunostaining was carried out by replacing the primary Ab with non-immune rabbit serum.

**Table 2** Sequences of the primers used in our RT-PCR experiments to detect RET/PCR rearrangements

| | Sequence | Localization |
|---|---|---|
| c-ret primers | | |
| tm1 (outer forward) | 5′-CTG TCC TCT TCT CCT TCA TC-3′ | exon 11, nt 1853–1872 |
| retc2 (outer reverse) | 5′-TGC AGG CCC CAT ACA ATT TG-3′ | exon 13, nt 2295–2314 |
| 26 (in-nested forward) | 5′-TCC ATG GAG AAC CAG GTC TCC-3′ | exon 11, nt 2026–2046 |
| RET/TK (in-nested reverse) | 5′-CTT TCA GCA TCT TCA CGG-3′ | exon 12, nt 485–493 |
| H4 primers | | |
| RET/PTC1 (outer forward) | 5′-ATT GTC ATC TCG CCG TTC-3′ | H4 exon 1, nt 196–213 |
| PTCI (in-nested) | 5′-AGA TAG AGC TGG AGA CCT AC-3′ | H4 exon 1, nt 272–291 |
| RIα primers | | |
| RET/PTC2 (outer forward) | 5′-TAT CGC AGG AGA GAC TGT GAT-3′ | RIα, nt 483–503 |
| PTC II (in-nested forward) | 5′-AGG GAG CTT TGG AGA ACT TG-3′ | RIα, nt 600–619 |
| ELE1 primers | | |
| PTC III (outer forward) | 5′-CAT GCC AGA GCA GAA GTC A-3′ | ELE1, nt 634–652 |
| RET/PTC3 (in-nested forward) | 5′-AAG CAA ACC TGC CAG TGG-3′ | ELE1, nt 697–714 |

*© 2001 Cancer Research Campaign*

### RESULTS

### Research of RET/PTC rearrangements in tumoural and nontumoural tissues infiltrated by lymphocytes

RET/PTC1 transcripts were detected in all the papillary carcinomas of family 1, whereas all the non-tumoural tissues presenting lymphocytic infiltration, and the adenoma from one of the members of this family, were negative (Figure 2A). In this family, all the samples were negative for RET/PTC2 and PTC3 (data not shown).

Concerning family 2, our data showed a strong band of RET/PTC3 positivity in all the papillary carcinomas. In the non-tumoural tissues infiltrated by lymphocytes, a band of positivity was also observed with an intensity varying slightly between the samples representative of the different members of the family. The intensity of the RET/PTC2 bands in the thyroiditis samples was, in all the cases, lower than in the tumours (Figure 3A). These samples were negative for RET/PTC1 and PTC2 (data not shown).

All the sporadic PTCs used as controls were negative for RET/PTC1, PTC2 and PTC3 (data not shown).

We also studied by RT-PCR the expression of the TK domain of *ret* in the different samples of both families. Expression of the *ret* TK domain is not normally detected in the thyroid gland, because the gene is only expressed in parafollicular C cells, which constitute about 1% of the normal thyroid cellular population (Suárez, 1998a). In our experiments we observed that in family 1, there was expression of the *ret* TK domain in the samples corresponding to the 3 papillary carcinomas. Surprisingly, a faint band of expression was also observed in the non-tumoural thyroid tissues infiltrated by lymphocytes of the patients bearing a carcinoma. The adenomatous tissue and its corresponding normal control were negative (Figure 2C). In samples of family 2, *ret* TK expression was detected in all carcinomatous as well as thyroiditis samples. The bands of positivity were weaker in the latter tissues (Figure 3C). As expected, *ret* TK expression was not detected in the normal unrelated control tissues (see the figures).

With the aim to establish whether or not the *ret* rearrangements were somatic or germinal, we also studied by RT-PCR RNAs extracted from the peripheral blood samples of 3 of the members of family 2 (patients 5, 6 and 7) positive for RET/PTC3, as well as genetic material prepared from a thyroid dystrophy diagnosed in one of their cousins. The data were negative (data not shown).

To confirm that the negative results were due neither to RNA degradation nor to a failure of extraction, a similar length of RNA transcript from the ubiquitously expressed β-actin gene was used as a reported target for amplification. As shown in the figures, an actin signal (72 bp long) was observed with a similar intensity in all the samples studied (Figures 2, 3B, D). Moreover, to eliminate all possibility of PCR contamination, a sample without RNA was added to all the experiments (see the figures).

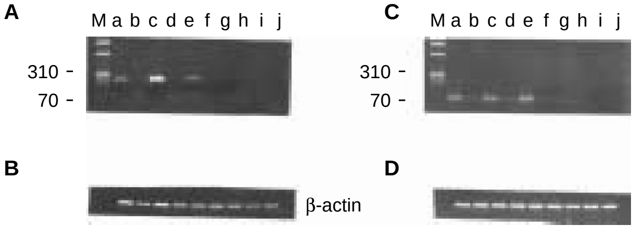

**Figure 2** Research by RT-PCR in members of family 1 of: (**A**) *ret* rearrangements. Lanes: a, c, and e: presence of RT/PTC 1 in the PTCs from cases 1, 2 and 3; b, d, f and h: absence of RET/PTC in thyroiditis from cases 1, 2, 3 and 4; g and i: absence of RET/PTC in the follicular adenoma from case 4 and in normal thyroid tissue; j: PCR reaction in the absence of genetic material. (**C**) *ret* TK domain expression. Lanes: a, c and e: in PTCs from cases 1, 2 and 3; b, d, f, and h: in thyroiditis from cases 1, 2, 3 and 4; g and i: in the follicular adenoma from case 4 and in normal thyroid tissue; j: PCR reaction in the absence of genetic material. (**B**, **D**) β-actin control of quantity and quality of genetic material. Ethidium bromide-stained 2% agarose gels of second round PCR products. M: molecular weight marquer ØX 174/Hae III

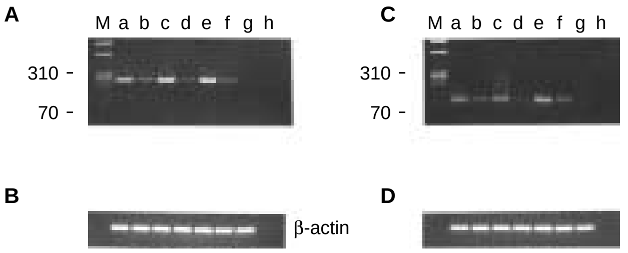

**Figure 3** Research by RT-PCR in members of family 2 of: (**A**) *ret* rearrangements. Lanes: a, c and e: presence of RET/PTC 3 in the PTCs from cases 5, 6 and 7; b, d and f: presence of RET/PTC 3 in thyroiditis from cases 5, 6 and 7; g: normal thyroid tissue; h: PCR reaction in the absence of genetic material. (**C**) *ret* TK domain expression. Lanes: a, c and e: in PTCs from cases 5, 6 and 7; b, d and f: in thyroiditis from cases 5, 6 and 7; g: normal thyroid tissue; h: PCR reaction without genetic material. (**B**, **D**) β-actin control of quantity and quality of genetic material. Ethidium bromide-stained 2% agarose gels of second round PCR products. M: molecular weight marquer ØX 174/Hae III

### Immunohistochemistry

The calcitonin labelling does not show C cell hyperplasia in any of the tissues studied (data not shown).

The polyclonal *ret* Ab gave a faint diffuse cytoplasmic labelling in cases 1, 2 and 3 in carcinomatous areas (Figure 4). In non-carcinomatous inflammatory areas, a faint and heterogeneous, cytoplasmic and apical labelling was observed, being noticed in some areas and absent in others (Figure 5).

In cases 5 and 7 (Figure 6), a strong apical labelling of the carcinomatous cells appeared in some areas. This apical labelling was weaker in case 6 (data not shown). In these 3 cases, the epithelial cells labelling of LT areas was unsteady, with cytoplasmic or apical labelling (data not shown).

No immunohistochemical labelling was observed in the adenoma (case 4).

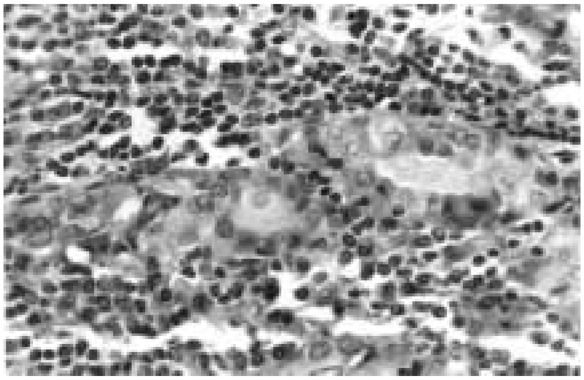

**Figure 4** Diffuse cytoplasmic labelling in a PTC of follicular pattern (case 2), using a polyclonal *ret* antibody (1/200) (magnification × 300). Lymphoplasmacytic infiltrate in the stroma is also stained

 *© 2001 Cancer Research Campaign*

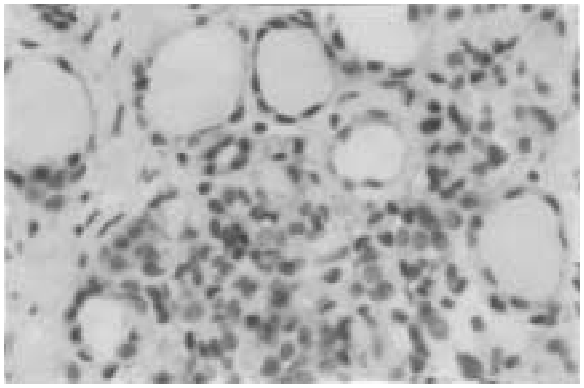

**Figure 5** Faint apical labelling of epithelial cells in LT area, without carcinomatous invasion (polyclonal *ret* antibody 1/200, magnification × 300, case 2)

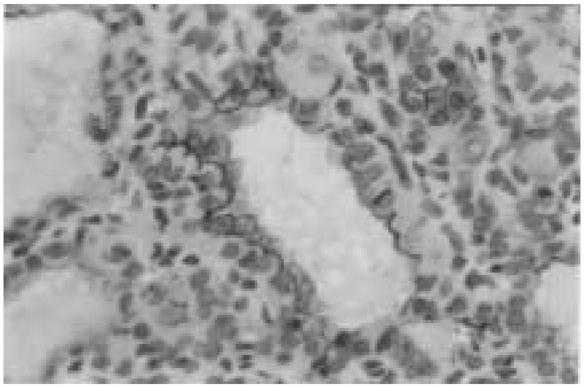

**Figure 6** Dense apical staining in a PTC of follicular pattern (polyclonal *ret* antibody 1/200, magnification × 400, case 7)

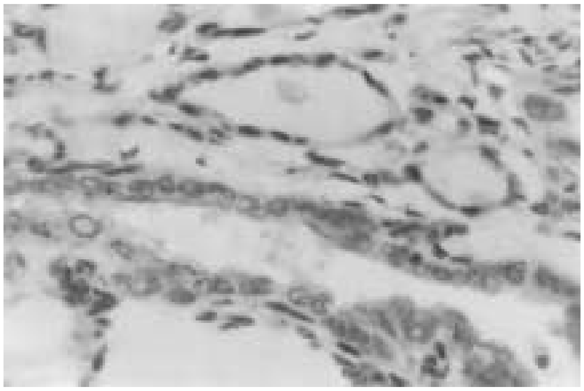

**Figure 7** Dense apical staining in a PTC of follicular pattern. Several unlabelled non-tumoural follicles are shown (polyclonal *ret* antibody 1/200, magnification × 400, case 5)

A faint and focal apical labelling was observed in one of the 4 sporadic control PTCs without RET/PTC rearrangement. All the other control sporadic PTCs were negative. In all the patients, the parenchyma without carcinoma and without LT was unstained (Figure 7).

### DISCUSSION

We looked for the eventual presence of *ret* proto-oncogene alterations, in members of 2 families presenting thyroid tumours, in majority of the papillary carcinoma type. Our results were compared with data obtained by screening non-tumoural thyroid tissue infiltrated by lymphocytes collected from the same patients, unrelated normal thyroid tissue and 4 unrelated sporadic papillary carcinomas.

In our study, carried out using the RT-PCR technique, all the PTCs presented a RET/PTC-activating rearrangement, from the PTC1 type in family 1 and from the PTC3 type in family 2. The follicular adenoma of one of the members of family 1 was negative. As for the non-tumoural thyroid tissues infiltrated by lymphocytes, the data were different in the 2 families. Whereas in family 1 all the samples were negative, in family 2 they were positive, but weaker than in the respective carcinomatous samples. It is interesting to point out that, when the degree of expression of the TK domain of *ret* was studied in thyroiditis or malignant thyroid tissues of both families, a variable *ret* activation was identified in all the samples by the presence of a PCR product of the predicted size. The exceptions were the adenoma of one of the members of family 1 and the control normal thyroid tissues, in which *ret* expression was not detected.

In family 2, as expected because of the detection in these samples of RET/PTC3 transcripts, we found the presence of *ret* expression in thyroid tissues infiltrated by lymphocytes. Unexpectedly in family 1 we found the presence of *ret* expression in thyroid tissues infiltrated by lymphocytes, while 3 independent experiments to detect the presence of RET/PTC chimeric cDNAs had been negative. Moreover we did not find the presence of tumour cells nor C cell hyperplasia in these tissues. The signal of *ret* TK domain expression being very weak in the thyroiditis samples of family 1, we can hypothesize a RET/PTC1 rearrangement in a minority of ‘normal’ thyrocytes, below the threshold of detection in our RT-PCR experiments. Another hypothesis might be the possible presence of rare isolated tumour cells in lymphocytic thyroiditis undetected by histology.

In PTC areas, the results of immunohistochemistry and RT-PCR correlated: the neoplastic cells of the 3 RET/PTC1-positive tumours and those of the 3 RET/PTC3-positive tumours displayed a distinct labelling. However, this labelling was diffuse and cytoplasmic in family 1 and apical in family 2. The diffuse cytoplasmic labelling type has already been reported in cases of PTC with RET/PTC1 activation (Sugg et al, 1998). Moreover, this type of labelling was found in PTC associated with familial adenomatous polyposis (Cetta et al, 1998), and has also been described in occult PTC (Viglietto et al, 1995). In our study, the faint labelling may be related to a low expression of *ret* protein by the RET/PTC1 oncogene as previously described by Jhiang et al (1996). Different levels of *ret* expression have been described with an intracytoplasmic or membranous localization. Recently, a membranous *ret* immunolabelling was described in 20 out of 99 PTCs presenting an overexpressed apparently wild-type *ret* gene, whereas a diffuse cytoplasmic labelling was observed in tumours with RET/PTC1, 2, 3 or 4 rearrangements (Ishizaka et al, 1992; Mayr et al, 1999). However, staining in PTC associated with a RET/PTC3 rearrangement has been reported infrequently and the different *ret* products have been described as localized in different cell compartments (Ishizaka et al, 1992).

In the LT areas, interpretation of immunohistochemical staining is difficult because of variable results. This interpretation is also hampered by a possible unrecognized tumour cell extension and by the *ret* expression of the lymphoplasmacytic infiltrate (Nakayama et al, 1999). With such restrictions, our findings suggest however

*© 2001 Cancer Research Campaign* 

an immunohistochemical *ret* protein expression in a few non-carcinomatous follicular epithelial cells in LT areas in both families 1 and 2, without clear-cut correlation between *ret* rearrangement and immunohistochemical *ret* protein expression. These data could be explained by a low abnormal expression of the wild-type *ret* gene as suggested by recent data of Bunone et al (2000) or, alternatively, by the presence of unknown forms of *ret* rearrangements. The expression of *ret* protein in the LT areas was shown in a recent publication (Sugg et al, 1998). Indeed, among 3 non-tumoural tissues with *ret* rearrangement studied by these authors, one case expressed a *ret* immunoreactivity in atypical epithelial cells associated with thyroiditis.

Our present data agree with results of previous publications (Suárez, 1998a, b) showing that *ret* activation in 'spontaneous' tumours is confined to papillary carcinomas. However, the presence of the same type of RET/PTC rearrangement in the tumoural genetic material of family 1 and tumoural and thyroiditis genetic material of members of family 2, raises several questions: whether or not (1) the mutation is germinal; (2) the members of these families are carriers of a common 'fragile' region in one (or both) of the genes participating at the formation of the RET/PTC chimeric genes, which may be the preferential target of different unknown genotoxic agents giving rise to the rearrangement or (3) the so-called 'spontaneous' tumours of these patients are the consequence of inadvertent exposure to lower doses of radiation or other genotoxic agents.

Our data showing the absence of *ret* rearrangements in blood samples of 3 of the members of family 2 presenting a RET/PTC3 chimeric gene in their tumours, as well as in the genetic material prepared from a thyroid dystrophy diagnosed in one of their cousins, suggest that the mutation is somatic.

In regard to the second question, we are unable to verify it because we do not have enough biological material to study by XL-PCR the genomic sequences where the break-points are generally located in the different RET/PTC rearrangements known until present (Grieco et al, 1990; Bongarzone et al, 1993; Santoro et al, 1994; Fugazzola et al, 1996; Bounacer et al, 1997; Suárez, 1998b). Finally, concerning the third question, there was no evidence in the clinical history of our patients of a possible low-dose irradiation and/or a chemical agent aggression. If the latter possibility cannot be completely discarded, the second hypothesis remains the most plausible one. Activating rearrangements of *ret* and other genes with TK activity (i.e. *trk* and *met*) are the consequence mainly of radiation and more rarely of chemical mutagens or perhaps other unknown genotoxic agents (Michelin et al, 1993; Sarasin et al, 1999). We can assume that the action of one of these genotoxic agents acting on a common 'fragile' site carried by our patients in one (or both) of the genes constituting the RET/PTC oncogene, may be at the origin of the rearrangements observed.

Our study is the first to demonstrate the presence of the same RET/PTC gene and overexpression of the tyrosine kinase domain of *ret*, in the papillary carcinomas of members of 2 unrelated families: in one case PTC1 and in the other PTC3. Moreover, our present and previous data (Bounacer et al, 1997) as well as data from different laboratories (Viglietto et al, 1995; Santoro et al, 1996; Wirtschafter et al, 1997; Cho et al, 1999) confirm that RET/PTC oncogenic activation is an early event of thyroid epithelial tumorigenesis.

In conclusion, altogether our data suggest that: (1) we are in the presence of familial PTCs in which RET/PTC may play a role of 'marker'. However, this marker seems to be particular to each family rather than a common one; (2) RET/PTC rearrangements are early events in PTC development, being already present in the 'normal' thyrocytes of the regions infiltrated by lymphocytes; and (3) these mutations may be the consequence of, until now, unknown genetic and/or microenvironmental factors, at least in our patients. Moreover, our results agree with those of Okayasu et al (1995) suggesting that lymphocytic thyroiditis is not only frequently associated to papillary carcinoma but is perhaps a predisposing factor for tumour development, as suggested by the presence of the same RET/PTC rearrangement in both tumoural and inflammatory tissues in the same patient.

### ACKNOWLEDGEMENTS

The authors are especially indebted to Dr B Franc and Dr B Caillou for helpful criticisms and to Dr Y Guillard and B Margoloff who performed the surgery of the patients.

Work in the laboratory of HG Suarez (UPR 2169 CNRS) was supported by grants from CNRS, ARC, EDF, Ligue Nationale Contre le Cancer, Fondation de France and Ministère de l'Education et de la Recherche.

### REFERENCES

Bongarzone I, Pierotti M, Monzini N, Mondellini P, Manenti G, DonghiS R, Pilotti S, Grieco M, Santoro M, Fusco A, Vecchio G and Della-Porta G (1989) High frequency of activation of tyrosine kinase oncogenes in human papillary thyroid carcinoma. *Oncogene* **4**: 1457–1462

Bongarzone I, Monzini N, Borrello MG, Carcano C, Ferraresi G, Arighi E, Mondellini P, Della-Porta G and Pierotti MA (1993) Molecular characterization of a thyroid tumor-specific transforming sequence formed by the fusion of ret tyrosine kinase and the regulatory subunit RIα of cyclic AMP-dependent protein kinase A. *Mol Cell Biol* **13**: 358–366

Bounacer A, Wicker R, Caillou B, Cailleux AF, Sarasin A, Schlumberger M and Suárez HG (1997) High prevalence of activating ret proto-oncogene rearrangements, in thyroid tumors from patients who had received. *Oncogene* **15**: 1263–1273

Bunone G, Uggeri M, Mondellini P, Pierroti MA and Bongarzone I (2000) RET receptor expression in thyroid follicular epithelial cell-derived tumors. *Cancer Res* **60**: 2845–2849

Cetta F, Chiapetta G, Mellilo RM, Petracci M, Montalto G, Santoro M and Fusco A (1998) The RET/PTC1 oncogene is activated in familial adenomatous polyposis associated thyroid papillary carcinomas. *J Clin Edocrinol Metab* **83**: 1003–1006

Chappuis S, Geneste O, Pasini P, Lenoir G and Billaud B (1996) RET et GDNF: un récepteur orphelin trouve une famille nourricière. Médecine/Sciences **12**: 1408–1413

Cho JY, Sagartz JE, Capen CC, Mazzaferri EL and Jhiang SM (1999) Early cellular abnormalities induced by RET/PTC 1 oncogene in thyroid-targeted transgenic mice. *Oncogene* **18**: 3659–3665

Delvincourt C, Patey M, Flament JB, Suárez HG, Larbre H, Jardillier JC and Delisle MJ (1996) Ret and trk proto-oncogene activation in thyroid papillary carcinomas in French patients from the Champagne-Ardenne region. *Clinical Biochemistry* **29**: 267–271

Fisher DK, Groves MD, Thomas SJ and Johnson PC (1989) Papillary carcinoma of the thyroid: additional evidence in support of a familial component. *Cancer Invest* **7**: 323–325

Fugazzola L, Pierotti MA, Vigano E, Pacini F, Vorontsova TV and Bongarzone I (1996) Molecular and biochemical analysis of RET/PTC4 a novel oncogenic rearrangement between RET and ELE1 genes, in a post-Chernobyl papillary thyroid cancer. *Oncogene* **13**: 1093

Gaulier A, Fourcade C, Szekeres G and Pulik M (1994) Bone marrow one step fixation-decalcification in lowy FMA solution: an immunohistological and in situ hybridization study. *Pathol Res Pract* **190**: 1149–1161

Gorson D (1992) Familial papillary carcinoma of the thyroid. *Thyroid* **2**: 131–132

Grieco M, Santoro M, Berlingieri MT, Melillo RM, Donghi R, Bongarzone I, Pierotti MA, Della-Porta G, Fusco A and Vecchio G (1990) PTC is a novel rearranged form of the ret proto-oncogene and is frequently detected in vivo in human thyroid papillary carcinomas. *Cell* **60**: 557–563

 *© 2001 Cancer Research Campaign*

Ishizaka Y, Kobayashi S, Ushijima T, Hirohashi S, Sugimura T and Nagao M (1991) Detection of ret TPC/PTC transcripts in thyroid adenomas and adenomatous goiter by an RT-PCR method. *Oncogene* **6**: 1667–1672

Ishizaka Y, Shima H, Sugimara T and Nagao M (1992) Detection of phosphorylated RET/PTC oncogene product in cytoplasm. *Oncogene* **7**: 1441–1444

Jhiang SM (2000) The ret proto-oncogene in human cancers. *Oncogene* **19**: 5590–5597

Jhiang SM, Sagartz JE, Tong Q, Parker-thouburg J, Capen CC, Cho JY, Xing S and Ledend C (1996) Targeted expression of the ret/PTC 1 oncogene induces papillary thyroid carcinomas. *Endocrinology* **137**: 375–378

Klugbauer S and Rabes HM (1999) The transcription coactivator HTIF1 and a related protein are fused to the RET receptor Tyrosine Kinase in childhood papillary thyroid carcinomas. *Oncogene* **18**: 4388–4393

Klugbauer S, Jauch A, Lengfelder E, Demidchik E and Rabes HM (2000) A novel type of RET rearrangement (PTC8) in childhood papillary thyroid carcinomas and characterization of the involved gene (RFG8). *Cancer Res* **60**: 7028–7032

Lote K, Andersen K, Nordal E and Brennhood IO (1980) Familial occurrence of papillary thyroid carcinoma. *Cancer* **46**: 1291–1297

Mayr B, Potter E, Goretzki P, Rüschoff J, Ditmaier W, Hoang-Vu C, Dralle H and Brabant G (1999) Expression of wild type *ret*, RET/PTC and RET/PTC variants in papillary thyroid carcinoma in Germany. *Langenbeck's Arch Surg* **384**: 54–59

McKee RF, Krukowski ZH and Matheson NA (1993) Thyroid neoplasia coexistent with chronic lymphocytic thyroiditis. *Brit J Surg* **80**: 1303–1304

Michelin S, Daya-Grosjean L, Sureau F, Saïd S, Sarasin A and Suárez HG (1993) Characterization of a c-met proto-oncogene actived in human Xeroderma pigmentosum cells after treatment with N-methyl-N′-nitro-N-nitrosoguanidine (MNNG). *Oncogene* **8**: 1983–1991

Nakayama S, Iida K, Tsuzuki T, Iwashita T, Murakami H, Asai N, Iwata Y, Ichihara M, Ito S, Kawai K, Asai M, Kurokawa K and Takahashi M (1999) Implication of expression of GDNF/Ret signalling componentsin differentiation of bone marrow haemopoietic cells. *Br J Haematol* **105**: 50–57

Okayasu I, Fujiwara M, Hara Y, Tanaka Y and Rose NR (1995) Association of chronic lymphocytic thyroiditis and thyroid papillary carcinoma. *Cancer* **76**: 2312–2318

Reda G, Cesareo R, Lolli E and Gargiulo A (1997) Thyroid cancer and Hashimoto's thyroiditis. *Minerva Chir* **52**: 139–141

Ron E, Kleinerman RA, Livolsi VA and Fraumeni JF (1991) Familial nonmedullary thyroid cancer. *Oncology* **48**: 309–311

Santoro M, Dathan N, Berlingieri MT, Bongarzone I, Paulin C, Grieco M, Pierotti MA, Vecchio G and Fusco A (1994) Molecular characterization of RET/PTC3; a novel rearranged version of the RET proto-oncogene in human thyroid papillary carcinoma. *Oncogene* **9**: 509–516

Santoro M, Chiapetta G, Cerrato A, Salvatore D, Zhang L, Manzo G, Picone A, Portella G, Santelli G, Vecchio G and Fusco A (1996) Development of thyroid papillary carcinomas secondary to tissue-specific expression of the RET/PTC1 oncogene in transgenic mice. *Oncogene* **12**: 1821–1826

Sarasin A, Bounacer A, Lepage F, Schlumberger M and Suárez HG (1999) Mechanisms of mutagenesis in mammalian cells: application to human thyroid tumors. *C. R. de l'Acad des Sciences* **322**: 143–149

Sclafani AP, Valdes M and Cho H (1993) Hashimoto's thyroiditis and carcinoma of the thyroid: optimal management. *Laryngoscope* **130**: 845–849

Soh EY and Parc S (1993) Diagnostic approach to thyroid carcinoma in Graves' disease. *Yousei Med J* **34**: 191–194

Soravia C, Sugg SL, Berk T, Mitri A, Cheng H, Gallinger S, Cohen Z, Asa SL and Bapat BV (1999) Familial adenomatous polyposis-associated thyroid cancer. *Am J Pathol* **154**: 127–135

Suárez HG (1998 a): La tumorigenèse épithéliale thyroïdienne associée aux radiations. Médecine/Sciences **14**: 1356–1365

Suárez HG (1998 b) Genetic alterations in epithelial human thyroid tumours. Clinical Endocrinology **48**: 531–546

Sugg S, Ezzat S, Rosen IB, Freeman JL and Asa SL (1998) Distinct multiple RET/PTC gene rearrangements in multifocal papillary thyroid neoplasia. *J Clin Endocrinol Metab* **83**: 4116–4122

Takahashi M, Buma Y, Iwamoto T, Inaguma Y, Ikeda H and Hiai H (1988) Cloning and expression of the ret proto-oncogene encoding a tyrosine kinase with two potential transmembrane domains. *Oncogene* **3**: 571–578

Viglietto G, Chiappetta G, Martinez-Tello FJ, Fukunaga FH, Tallini G, Rigopoulou D, Visconti R, Mastro A, Santoro M and Fusco A (1995) RET/PTC oncogene activation is an early event in thyroid carcinogenesis. *Oncogene* **11**: 1207–1210

Walker RP and Paloyan E (1990) The relationship between Hashimoto's thyroiditis, thyroid neoplasia and primary hyperparathyroidism. *Otolaryngol Clin North Am* **23**: 291–302

Wirtschafter A, Schmidt R, Rosen D, Kundu N, Santoro M and Fusco A (1997) Expression of RET/PTC fusion gene as a marker for papillary carcinoma in Hashimoto's thyroiditis. *Laryngoscope* **107**: 95–100

© 2001 Cancer Research Campaign
